# Impact of Long-Term Cannabidiol (CBD) Treatment on Mouse Kidney Transcriptome

**DOI:** 10.3390/genes15121640

**Published:** 2024-12-21

**Authors:** Mikołaj Rokicki, Jakub Żurowski, Sebastian Sawicki, Ewa Ocłoń, Tomasz Szmatoła, Igor Jasielczuk, Karolina Mizera-Szpilka, Ewelina Semik-Gurgul, Artur Gurgul

**Affiliations:** 1 Department of Basic Sciences, Faculty of Veterinary Medicine, University of Agriculture in Kraków, Redzina 1C, 30-248 Krakow, Poland; mikolaj.rokicki@student.urk.edu.pl (M.R.); kubazurowski@gmail.com (J.Ż.); tomasz.szmatola@urk.edu.pl (T.S.); igor.jasielczuk@urk.edu.pl (I.J.); 2 Department of Animal Reproduction, Anatomy and Genomics, University of Agriculture in Kraków, Mickiewicza 24/28, 30-059 Krakow, Poland; bastian.sawicki@gmail.com; 3 Laboratory of Recombinant Proteins Production, Faculty of Veterinary Medicine, University of Agriculture in Kraków, Redzina 1C, 30-248 Krakow, Poland; ewa.oclon@urk.edu.pl; 4 Department of Infectious Diseases and Public Health, Faculty of Veterinary Medicine, University of Agriculture in Kraków, Redzina 1C, 30-248 Krakow, Poland; karolina.mizera@urk.edu.pl; 5 Department of Animal Molecular Biology, National Research Institute of Animal Production, Krakowska 1, 32-083 Balice, Poland; ewelina.semik@iz.edu.pl

**Keywords:** cannabidiol, CBD, RNA-seq, mouse model, kidney

## Abstract

Background: Cannabidiol, which is one of the main cannabinoids present in *Cannabis sativa* plants, has been shown to have therapeutic properties, including anti-inflammatory and antioxidant effects that may be useful for treatment of various kidney conditions. Objectives: This article investigates the effect of long-term cannabidiol (CBD) treatment on changes in the renal transcriptome in a mouse model. The main hypothesis was that systematic CBD treatment would affect gene expression associated with those processes in the kidney. Methods: The study was conducted on male C57BL/6J mice. Mice in the experimental groups received daily intraperitoneal injections of CBD at doses of 10 mg/kg or 20 mg/kg body weight (b.w.) for 28 days. After the experiment, kidney tissues were collected, RNA was isolated, and RNA-Seq sequencing was performed. Results: The results show CBD’s effects on changes in gene expression, including the regulation of genes related to circadian rhythm (e.g., *Ciart*, *Nr1d1*, *Nr1d2*, *Per2*, and *Per3*), glucocorticoid receptor function (e.g., *Cyp1b1*, *Ddit4*, *Foxo3*, *Gjb2*, and *Pck1*), lipid metabolism (e.g., *Cyp2d22*, *Cyp2d9*, *Decr2 Hacl1*, and *Sphk1*), and inflammatory response (e.g., *Cxcr4* and *Ccl28*). Conclusions: The obtained results suggest that CBD may be beneficial for therapeutic purposes in treating kidney disease, and its effects should be further analyzed in clinical trials.

## 1. Introduction

Cannabidiol (CBD) is one of the most common cannabinoids extracted from *Cannabis sativa* [1]. It belongs to the class of terpenophenolic compounds and is represented by the C_21_H_30_O_2_ chemical formula [2]. Most current research into the medicinal values of cannabidiol indicates that it has anti-inflammatory, antioxidant, anxiolytic, antipsychotic, and analgesic properties [3]. The mechanism of action of CBD is complex and multi-dimensional. Briefly, CBD interacts with the endocannabinoid system, showing low affinity for cannabinoid receptors CB1 and CB2, and acts as an inverse agonist for CB2 [4]. CBD affects ion channels, most notably by potentiation of the activity of hyperpolarization-activated cyclic nucleotide-gated channels—HCN4 channels—which are important in physiological functions, including cardiac pacemaker function [5]. Furthermore, it modulates synaptic coordination by inhibiting the pro-excitatory effects of lysophosphatidylinositol (LPI) at GPR55 receptors (G protein-coupled receptor 55), thus reducing hyperexcitability and seizure activity [6]. CBD also activates 5-HT1A (serotonin 1A) receptors, which are associated with its anxiolytic and antiepileptic effects [7]. Moreover, CBD interacts with the transient receptor potential (TRP) channels, especially TRPV1-4 and TRPA1, contributing to its diverse pharmacological effects, including anti-inflammatory and analgesic effects [8].

Some available studies have also revealed that CBD has impacts on renal functions and could be beneficial in the treatment of certain kidney disorders [9]. It has been suggested that inhibition of the CB1 receptor may improve renal function in both chronic and acute models of kidney injury through decreased levels of blood urea nitrogen, serum creatinine, and albuminuria. On the other hand, activation of CB2 receptors showed a promising effect in attenuating renal impairment in terms of a decrease in serum creatinine and albuminuria levels [10]. In addition, CBD has shown protective effects regarding doxorubicin-induced nephropathy, with improvements in markers of oxidative stress and inflammatory reaction in multiple animal models [11]. Other studies suggest that cannabinoids could additionally offer therapeutic potential for managing symptoms associated with renal failure, such as inflammation, pain, and nausea [9].

A look into the extant literature shows that the effects of CBD on various organs and physiological mechanisms are complex and not fully understood, especially at the molecular level [12]. Thus, the present study aimed to evaluate the long-term effect of CBD treatment on transcriptional profiles in renal tissues using a mouse model.

The principal hypothesis of the study was that systematic administration of CBD would result in remarkable changes in gene expression in the kidney, allowing for the identification of specific genes and molecular pathways related to the organism’s response to CBD and, therefore, a better understanding of its therapeutic implications in renal function.

## 2. Material and Methods

The experiment involved 12 male mice of the C57BL/6J strain, starting at an age of 60 days. Throughout the experiment, the mice were kept at a temperature of 22 °C (±2 °C) and a humidity level of 55% (±10%), with a light cycle of 12 h of light and 12 h of darkness. Mice were provided with ad libitum access to food and water. This study included three groups of mice, each consisting of 4 individuals. The experimental groups received a daily intraperitoneal injection of a CBD solution (200 μL) for 28 days. The CBD was dissolved in saline with 2% Tween 80, and the groups differed in CBD dosage, receiving either 10 mg/kg or 20 mg/kg of body weight (b.w.). The control group received saline (200 μL) with 2% Tween 80.

After the 28-day period, the mice were euthanized using inhalation anesthesia with isoflurane, followed by spinal cord transection. All animal procedures were reviewed and approved by the II Local Ethics Committee in Kraków (permission number 90/2022). After euthanasia, kidneys were collected for RNA isolation using Trizol reagent (Thermo Fisher, Waltham, MA, USA). The quality of the obtained RNA was assessed using TapeStaion 4150 (Agilent, Santa Clara, CA, USA) analysis and RNA integrity number evaluation (RIN > 8). The Poly(A) RNA Selection Kit (Lexogen, Wien, Austria) was used to purify RNA fragments containing polyadenine (poly(A)) tails. For library preparation, 50 ng of mRNA and the CORALL RNA-Seq V2 Library Prep Kit with UDIs (Lexogen, Wien, Austria) were used. The resulting libraries underwent quality assessment and were sequenced commercially with paired-end 2 × 150 bp reads on a NovaSeq6000 system (Illumina, San Diego, CA, USA), generating datasets with a minimum of 28 million reads per sample.

Quality control of raw reads was performed using FastQC software (v0.11.9). Sequence trimming and filtering were carried out using Flexbar software (3.5.0) [13]. The filtered reads were then mapped to the mouse GRCm39 reference genome using STAR software (2.7.5c) [14]. Read counts were obtained using Htseq-count software (1.99.2) [15]. Normalization and differential expression analyses were conducted using DESeq2 software [16] implemented in iDEP2.0 (integrated Differential Expression and Pathway analysis, v2.01) [17]. iDEP2.0 was also used for analyzing expression profile diversity through Principal Components Analysis (PCA) and hierarchical clustering based on Euclidean distance. Additionally, the software assessed the overrepresentation of differentially expressed genes in biological processes from the Gene Ontology (GO) database [18], as well as KEGG (Kyoto Encyclopedia of Genes and Genomes) pathways, for visualization purposes only. Fisher’s exact test was applied for this purpose, using all known mouse genes as the background, and genes or processes with a corrected *p*-value (Benjamani–Hochberg correction) of <0.1 were considered significant.

Raw reads, as well as raw read counts, were deposited in the Gene Expression Omnibus (GEO) and Sequence Read Archive (SRA) databases from the National Center for Biotechnology Information (NCBI) under the accession number GSE275998.

The results of RNA-Seq were validated for four selected genes (*Ypel2*, *Dbp*, *Npas2*, and *Acmsd*) affected in at least one treatment using quantitative real-time PCR. The *Mrpl32* and *Ppia* genes were used as an endogenous control [19,20]. For cDNA synthesis, 500 ng of RNA was used with the High-Capacity cDNA Reverse Transcription Kit (Thermo Fisher Scientific), following the manufacturer’s instructions. RT-qPCR was performed using the AmpliQ 5× HOT EvaGreen^®^ qPCR Mix Plus (ROX) kit (Novazym, Poznań, Poland) and primers designed to target mRNA sequences spanning two adjacent exons (sequences included in Supplementary File S1). Each sample was analyzed in triplicate on the QuantStudio 7 Flex system (Thermo Fisher Scientific). Relative gene expression levels were calculated using the ΔΔCt method.

To evaluate concordance in relative expression levels between RNA-Seq and RT-qPCR methods, correlation coefficient analysis was performed using mean expression values within groups. Statistical calculations were conducted with JASP 0.11.1 software. The Shapiro–Wilk test was used to assess data distribution, and the Spearman correlation coefficient was applied for the analysis due to the non-normal distribution of the data.

## 3. Results

**Figure 1:**
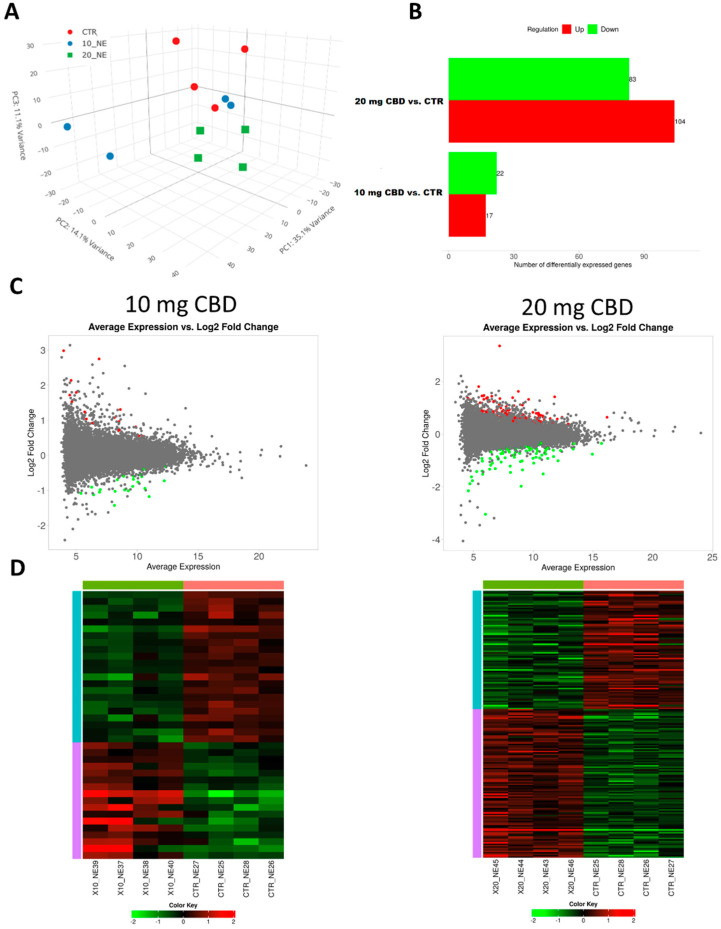
Results of RNA-Seq data analysis. (**A**) Principal components analysis for all samples; (**B**) number of genes altered with both CBD treatments; (**C**) MA plots; (**D**) heatmaps for genes significantly altered using CBD treatments in kidneys.

The number of uniquely mapped reads ranged from approximately 19.5 to 28.7 million (M), with an average of 22.1 M (±2.9 SD M) per sample (Supplementary File S2). Based on the normalized reads mapped to genes, a diversity analysis of expression profiles was conducted using PCA. This analysis revealed a relatively low level of variation in expression profiles between the study groups (Figure 1). The observed dispersion of samples suggests that those in both CBD-treated groups exhibit moderate changes in their transcriptional profiles compared to the control group, with these differences being particularly evident in the third PCA component (Figure 1). This suggests that CBD induces only minor changes in gene expression in the kidney, which are detectable primarily through detailed comparative analysis.

The comparative analysis involved evaluating gene expression differences between the control and treated groups. In the group treated with 10 mg CBD/kg b.w., 17 genes (43.6%) showed increased expression, while 22 genes (56.4%) showed decreased expression. In the group treated with 20 mg CBD/kg b.w., 104 genes (55.6%) exhibited increased expression, and 83 genes (44.4%) exhibited decreased expression (Figure 1; Table 1; Supplementary File S3). These results suggest a clear dose-dependent effect of CBD on the gene expression profile. However, the Venn diagram analysis shows that, of the 22 genes downregulated with the 10 mg CBD/kg b.w. treatment, 20 were also downregulated using a 20 mg CBD dose. Also, nearly half of the upregulated genes were common for both treatments, which suggests that the dose only affects strength, not the mechanisms of cellular response (Supplementary File S3).

The results of RNA-Seq validation with qPCR showed that the performed analysis was sufficiently accurate with high, and in most cases significant (*p* < 0.05, except *Npas2* gene for which *p* = 0.088), Spearman’s rank correlation coefficients in a range from 0.71 to 0.97 for individual genes (mean of 0.85) (Supplementary File S1).

Table 2 presents the results of over-representation tests for the altered genes in specific biological processes from the Gene Ontology database. A more detailed analysis, along with the underlying gene IDs, is presented in Supplementary File S4. Among the enriched processes (FDR < 0.05), ones associated with circadian rhythm cycling (including e.g., *Ciart*, *Nr1d1*, *Nr1d2*, *Per2*, and *Per3* genes), glucocorticoid receptor function (e.g., *Cyp1b1*, *Ddit4*, *Foxo3*, *Gjb2*, and *Pck1* genes), response to stimulus (e.g., *Ppard*, *Hmox1*, *Bmal1*, and *Gatm* genes), and metabolic processes (e.g., *Cyp2d22*, *Cyp2d9*, *Decr2 Hacl1*, and *Sphk1*) stand out in the foreground.

## 4. Discussion

In this study, we focus on the gene expression alterations stimulated in a mouse kidney by long-term intraperitoneal CBD administration, with special attention given to the protein-coding genes, without detailed analysis of long non-coding RNA that may also impact kidney health [21]. Our study uses a simple yet labor-intensive research model in which C57BL/6J mice were treated daily with three different CBD doses for 28 days. These treatments resulted in alterations of several gene expression levels that were further analyzed for their functional significance. The analysis of the performed over-representation test results indicated that CBD potentially influences many cellular processes in the kidney by regulating genes associated with circadian rhythm cycling, glucocorticoid receptor function, response to stimulus, and metabolic processes. Among the processes, we found the ones associated with the circadian clock and glucocorticoid receptor functioning as the most important from the point of view of clinical CBD applications.

The internal cellular circadian clock is present in nearly every cell in the body, and it contributes to the regulation of numerous processes related to internal changes in behavior and physiological functions that are coordinated by the internal molecular clock [22]. The circadian clocks in the kidney cells influence the daily rhythm of sodium, potassium, chloride, and phosphate excretion [23], and it was shown that the disruption of these daily patterns can be associated with hypertension and cardiovascular diseases [24,25]. Studies by Mohandas et al. (2022) [26] have demonstrated that urine excretion and urine pH also fluctuate in a daily cycle. Furthermore, it was suggested that peripheral circadian clocks in the kidneys affect the daily rhythm of blood pressure [23], potentially through the regulation of the secretion of hormones and the activity of enzymes involved in blood pressure regulation [27].

The available literature presents evidence that CBD administration influences the circadian genes in microglial cells, leading to the deregulation of circadian rhythms [28]. Additionally, cannabinoids, including CBD, have been found to interact with the brain’s circadian clock by modulating neuronal activity in the suprachiasmatic nucleus, which may explain the altered sense of time experienced by users of cannabinoid-containing products [29].

The results of the gene expression analysis performed in this study confirmed those results and indicated that both applied CBD doses stimulated the expression of several genes related to the regulation of the circadian cycle in the kidney. One, and potentially the most meaningful, of these genes was *Nr1d2* (Nuclear Receptor Subfamily 1 Group D Member 2), also known as *rev-erbβ*. This gene is a key regulator of circadian rhythms and a component of cellular metabolism [30]. As part of the biological clock, *Nr1d2* plays a significant role in synchronizing internal physiological processes with the daily cycle [31]. Rev-erbα is one of the main regulators of the negative feedback loop in the circadian clock. By repressing the expression of the *Bmal1* gene, Rev-erbα helps maintain the cyclical nature of activity and rest in clock genes, which is crucial for keeping biological rhythms in the proper phase [32]. The KEEG pathway associated with this gene and circadian rhythm regulation is presented in the Supplementary File S5.

The obtained results also reveal negative regulation of genes linked to the glucocorticoid receptor (GR) signaling pathway. This pathway is essential for maintaining the body’s homeostasis by regulating various physiological processes, such as the stress response, metabolism, and immune function [33]. Glucocorticoids, like cortisol or corticosterone in rodents, modulate the body’s response to environmental stressors and are clinically used as anti-inflammatory agents [34]. Glucocorticoid activity in the kidney is vital, as it supports the regulation of water and electrolyte balance, which is crucial for maintaining blood pressure [35].

The analysis of the results of this study reveal that among the altered genes responsible for the negative regulation of glucocorticoids action is, e.g., *Fkbp5* (FK506-binding protein 5). This gene acts as a negative regulator of glucocorticoid action. Its mechanism involves increasing cellular resistance to glucocorticoids, thereby reducing their effectiveness in response to stress [36]. In vitro experiments have shown that *Fkbp5* disrupts the interaction between the glucocorticoid receptor (GR) complex and the transport protein dynein, delays the nuclear translocation of GR, and decreases the transcriptional activity of the gene for GR [37]. Thus, CBD seems to have potential in the regulation of glucocorticoid action, which may have several implications for metabolic, anti-inflammatory, immune-suppressive, and neurologic effects [38].

Summarizing, the present study demonstrates a dose-dependent effect of CBD on the transcriptional profile of mouse kidneys and presents genes and processes affected by a long-term CBD treatment in this model organ. The processes associated with the altered genes included the regulation of circadian rhythm cycling and glucocorticoid receptor function, which are strong candidates for further research on this compound and its potential benefits in the treatment of renal disorders. The obtained results also suggest that higher doses of CBD may be more effective for therapeutic purposes in kidney-related conditions (as they stimulated more transcriptional changes); however, further investigations using clinical trials are needed to test this hypothesis.

## Figures and Tables

**Table 1:** The most significantly altered genes with both CBD treatments (top 12).

			10 mg CBD vs. Control		20 mg CBD vs. Control	
Symbol	Ensembl ID	Description	log2FC *	FDR **	log2FC	FDR **
Dbp	ENSMUSG00000059824	D site albumin promoter binding protein	2.75	9.29 × 10^−34^	3.36	4.79 × 10^−52^
Ppard	ENSMUSG00000002250	peroxisome proliferator activator receptor delta	−0.98	3.11 × 10^−10^	−1.03	1.30 × 10^−11^
Per3	ENSMUSG00000028957	period circadian clock 3	1.08	1.93 × 10^−7^	1.39	1.09 × 10^−13^
Npas2	ENSMUSG00000026077	neuronal PAS domain protein 2	−1.42	1.67 × 10^−5^	−1.45	4.00 × 10^−6^
Ppm1h	ENSMUSG00000034613	protein phosphatase 1H (PP2C domain containing)	−0.55	8.22 × 10^−5^	−0.56	1.63 × 10^−5^
Ypel2	ENSMUSG00000018427	yippee like 2	−1.01	1.00 × 10^−4^	−1.35	8.63 × 10^−10^
Acmsd	ENSMUSG00000026348	amino carboxymuconate semialdehyde decarboxylase	−0.76	1.05 × 10^−3^	−0.91	3.50 × 10^−6^
Rorc	ENSMUSG00000028150	RAR-related orphan receptor γ	−0.68	1.05 × 10^−3^	−0.57	7.06 × 10^−3^
Nfil3	ENSMUSG00000056749	nuclear factor. interleukin 3, regulated	−0.98	2.06 × 10^−3^	−1.31	6.30 × 10^−7^
Cyp2a5	ENSMUSG00000005547	cytochrome P450, family 2, subfamily a, polypeptide 5	1.42	4.59 × 10^−8^	1.42	4.59 × 10^−8^
Bhlhe41	ENSMUSG00000030256	basic helix-loop-helix family, member e41	1.46	1.45 × 10^−6^	1.46	1.45 × 10^−6^
	ENSMUSG00000120875	novel transcript	1.25	1.75 × 10^−6^	1.25	1.75 × 10^−6^

* log2 fold change in expression level; ** *p*-value of the Wald test (DeSeq2) for differential expression, corrected for multiple testing using the FDR method.

**Table 2:** List of top GO biological processes (FDR < 0.05) overrepresented by genes altered through both CBD treatments.

Biological Process	CBD Dose (mg/kg b.w.)	Gene Regulation	FDR *	Enrichment **
Response to stimulus	10	Up	0.049	72
Circadian rhythm	10	Up	0.049	23.1
Circadian behavior	10	Up	0.049	76.2
Nutrient response	10	Down	0.025	17.5
Response to redox states	10	Down	0.025	157.8
Negative regulation of the glucocorticoid receptor signaling pathway	10	Down	0.031	210.4
Diurnal regulation of gene expression	10	Down	0.031	32.7
Intracellular receptor signaling pathway	10	Down	0.031	12.4
Glucocorticoid receptor signaling pathway	10	Down	0.031	114.8
Corticosteroid receptor signaling pathway	10	Down	0.031	78.9
Physiological rhythm	20	Up	0.026	6.4
Response to the stimulus	20	Up	0.025	19.3
Circadian rhythm	20	Up	0.025	20.4
Metabolism process of arachidonic acid	20	Up	0.047	17.8
Cellular response to a corticosteroid stimulus	20	Up	0.015	10.8
Process of qualitative biological regulation	20	Up	0.010	1.8
Diurnal regulation of gene expression	20	Up	0.011	12.8
Cellular response to peptides	20	Up	0.010	4.7
Metabolism process of unsaturated fatty acids	20	Up	0.010	5.1
Cellular response to potassium ions	20	Up	0.015	95.2
Organic acid biosynthesis process	20	Down	0.031	5.9
Monosaccharide biosynthesis process	20	Down	0.031	4.3
The biosynthetic process of carboxylic acids	20	Down	0.031	5.9
Organic acid metabolism process	20	Down	0.036	3.2
The metabolism process of monocarboxylic acids	20	Down	0.010	3.9

* Fisher’s exact test probability value for the distribution of genes in individual pathways, adjusted for multiple testing using the FDR approach; ** fold enrichment indicates the extent (fold change) to which the genes tested are over-represented in each biological process compared to expectations for the whole genome.

## Data Availability

Raw reads, as well as raw read counts, were deposited in the Gene Expression Omnibus (GEO) and Sequence Read Archive (SRA) databases from the National Center for Biotechnology Information (NCBI) under the accession number GSE275998.

## Supplementary Materials

The following supporting information can be downloaded at: https://www.mdpi.com/article/10.3390/genes15121640/s1, Supplementary File S1. Methods, primes and results of qPCR analysis along with the result of correlation analysis between RNA-Seq and qPCR methods; Supplementary File S2. Sequencing and read mapping statistics; Supplementary File S3. Results of differential expression analysis and genes comparative analysis; Supplementary File S4. Top 20 GO biological processes overrepresented by genes altered by both CBD treatments. Supplementary File S5. Visualization of KEGG pathway associated with circadian rhythm regulation with marked (red) genes altered by 20 mg CBD treatment.

## References

[1] Burstein S. (2015). Cannabidiol (CBD) and its analogs: A review of their effects on inflammation. Bioorg. Med. Chem..

[2] Radwan M., Chandra S., Gul S., El Sholy M. (2021). Cannabinoids, phenolics, terpenes, and alkaloids of Cannabis. Molecules.

[3] Atalay S., Jarocka-Karpowicz I., Skrzydlewska E. (2019). Antioxidative and anti-inflammatory properties of cannabidiol. Antioxidants.

[4] Raïch I., Lillo J., Rivas-Santisteban R., Rebassa J.B., Capó T., Santandreu M., Cubeles-Juberias E., Reyes-Resina I., Navarro G. (2024). Potential of CBD acting on cannabinoid receptors CB1 and CB2 in ischemic stroke. Int. J. Mol. Sci..

[5] Page D.A., Ruben P.C. (2024). Cannabidiol potentiates hyperpolarization-activated cyclic nucleotide-gated (HCN4) channels. J. Gen. Physiol..

[6] Rosenberg E.C., Chamberland S., Bazelot M., Nebet E.R., Wang X., McKenzie S., Jain S., Greenhill S., Wilson M., Marley N. (2023). Cannabidiol modulates excitatory-inhibitory ratio to counter hippocampal hyperactivity. Neuron.

[7] Martínez-Aguirre C., Carmona-Cruz F., Velasco A.L., Velasco F., Aguado-Carrillo G., Cuéllar-Herrera M., Rocha L. (2020). Cannabidiol acts at 5-HT1A receptors in the human brain: Relevance for treating temporal lobe epilepsy. Front. Behav. Neurosci..

[8] Etemad L., Karimi G., Alavi M.S., Roohbakhsh A. (2022). Pharmacological effects of cannabidiol by transient receptor potential channels. Life Sci..

[9] Worth H., O’Hara D., Agarwal N., Collister D., Brennan F., Smyth B. (2022). Cannabinoids for symptom management in patients with kidney failure: A narrative review. Clin. J. Am. Soc. Nephrol..

[10] Zhao Z., Yan Q., Xie J., Liu Z., Liu F., Liu Y., Zhou S., Pan S., Liu D., Duan J. (2024). The intervention of cannabinoid receptor in chronic and acute kidney disease animal models: A systematic review and meta-analysis. Diabetol. Metab. Syndr..

[11] Soliman N.A., Dahmy SI E., Shalaby A.A., Mohammed K.A. (2024). Prospective affirmative therapeutics of cannabidiol oil mitigates doxorubicin-induced abnormalities in kidney function, inflammation, and renal tissue changes. Naunyn-Schmiedeberg’s Arch. Pharmacol..

[12] Naya N., Kelly J., Corna G., Golino M., Abbate A., Toldo S. (2023). Molecular and cellular mechanisms of action of cannabidiol. Molecules.

[13] Dodt M., Roehr J.T., Ahmed R., Dieterich C. (2012). FLEXBAR—Flexible barcode and adapter processing for next-generation sequencing platforms. Biology.

[14] Dobin A., Davis C.A., Schlesinger F., Drenkow J., Zaleski C., Jha S., Batut P., Chaisson M., Gingeras T.R. (2013). STAR: Ultrafast universal RNA-seq aligner. Bioinformatics.

[15] Anders S., Pyl P.T., Huber W. (2015). HTSeq—A Python framework to work with high-throughput sequencing data. Bioinformatics.

[16] Love M.I., Huber W., Anders S. (2014). Moderated estimation of fold change and dispersion for RNA-seq data with DESeq2. Genome Biol..

[17] Ge X. (2021). iDEP Web application for RNA-Seq data analysis. RNA Bioinformatics.

[18] Ge S.X., Son E.W., Yao R. (2018). iDEP: An integrated web application for differential expression and pathway analysis of RNA-seq data. BMC Bioinform..

[19] Muñoz J.J., Anauate A.C., Amaral A.G., Ferreira F.M., Watanabe E.H., Meca R., Ormanji M.S., Boim M.A., Onuchic L.F., Heilberg I.P. (2021). Ppia is the most stable housekeeping gene for qRT-PCR normalization in kidneys of three Pkd1-deficient mouse models. Sci. Rep..

[20] Secio-Silva A., Emrich F., Evangelista-Silva P.H., Prates R.P., Hijo A.H., Figueira-Costa T.N., Schaeffer M., Goulart-Silva F., Peliciari-Garcia R.A., Bargi-Souza P. (2023). Which housekeeping gene is the best choice for RT-qPCR analysis in mice fed with a high-fat diet? Studies in the liver, kidney, pancreas, and intestines. Gene Rep..

[21] Moreno J.A., Hamza E., Guerrero-Hue M., Rayego-Mateos S., García-Caballero C., Vallejo-Mudarra M., Metzinger L., Metzinger-Le Meuth V. (2021). Non-Coding RNAs in Kidney Diseases: The Long and Short of Them. Int. J. Mol. Sci..

[22] Costello H.M., Johnston J.G., Juffre A., Crislip G.R., Gumz M.L. (2022). Circadian clocks of the kidney: Function, mechanism, and regulation. Physiol. Rev..

[23] Stow L.R., Gumz M.L. (2011). The circadian clock in the kidney. J. Am. Soc. Nephrol. JASN.

[24] Goldman R. (1951). Studies in diurnal variation of water and electrolyte excretion; nocturnal diuresis of water and sodium in congestive cardiac failure and cirrhosis of the liver. J. Clin. Investig..

[25] Dyer A.R., Martin G.J., Burton W.N., Levin M., Stamler J. (1998). Blood pressure and diurnal variation in sodium, potassium, and water excretion. J. Hum. Hypertens..

[26] Mohandas R., Douma L.G., Scindia Y., Gumz M.L. (2022). Circadian rhythms and renal pathophysiology. J. Clin. Investig..

[27] Firsov D., Bonny O. (2018). Circadian rhythms and the kidney. Nat. Rev. Nephrol..

[28] Lafaye G., Desterke C., Marulaz L., Benyamina A. (2019). Cannabidiol affects circadian clock core complex and its regulation in microglia cells. Addict. Biol..

[29] Acuña-Goycolea C., Obrietan K., van den Pol A.N. (2010). Cannabinoids excite circadian clock neurons. J. Neurosci..

[30] Noh S.G., Jung H.J., Kim S., Arulkumar R., Kim D.H., Park D., Chung H.Y. (2022). Regulation of Circadian Genes Nr1d1 and Nr1d2 in Sex-Different Manners during Liver Aging. Int. J. Mol. Sci..

[31] Dumas B., Harding H.P., Choi H.S., Lehmann K.A., Chung M., Lazar M.A., Moore D.D. (1994). A new orphan member of the nuclear hormone receptor superfamily closely related to Rev-Erb. Mol. Endocrinol..

[32] Burris T.P. (2008). Nuclear hormone receptors for heme: REV-ERBα and REV-ERBβ are ligand-regulated components of the mammalian clock. Mol. Endocrinol..

[33] Wang J., Harris C. (2015). Glucocorticoid Signaling: From Molecules to Mice to Man.

[34] Vandewalle J., Luypaert A., De Bosscher K., Libert C. (2018). Therapeutic mechanisms of glucocorticoids. Trends Endocrinol. Metab..

[35] Mangos G., Whitworth J., Williamson P., Kelly J. (2003). Glucocorticoids and the kidney. Nephrology.

[36] Zannas A.S., Wiechmann T., Gassen N.C., Binder E.B. (2016). Gene–stress–epigenetic regulation of FKBP5: Clinical and translational implications. Neuropsychopharmacol. Rev..

[37] Wochnik G.M., Ruegg J., Abel G.A., Schmidt U., Holsboer F., Rein T. (2005). FK506-binding proteins 51 and 52 differentially regulate dynein interaction and nuclear translocation of the glucocorticoid receptor in mammalian cells. J. Biol. Chem..

[38] Chourpiliadis C., Aeddula N.R. (2024). Physiology, Glucocorticoids. StatPearls [Internet].

