# Supplementary figures and images for: Impact of Long-Term Cannabidiol (CBD) Treatment on Mouse Kidney Transcriptome
Source: Genes (Basel). 2024 Dec 21;15(12):1640. doi: 10.3390/genes15121640 (PMC11675924; doi:10.3390/genes15121640)

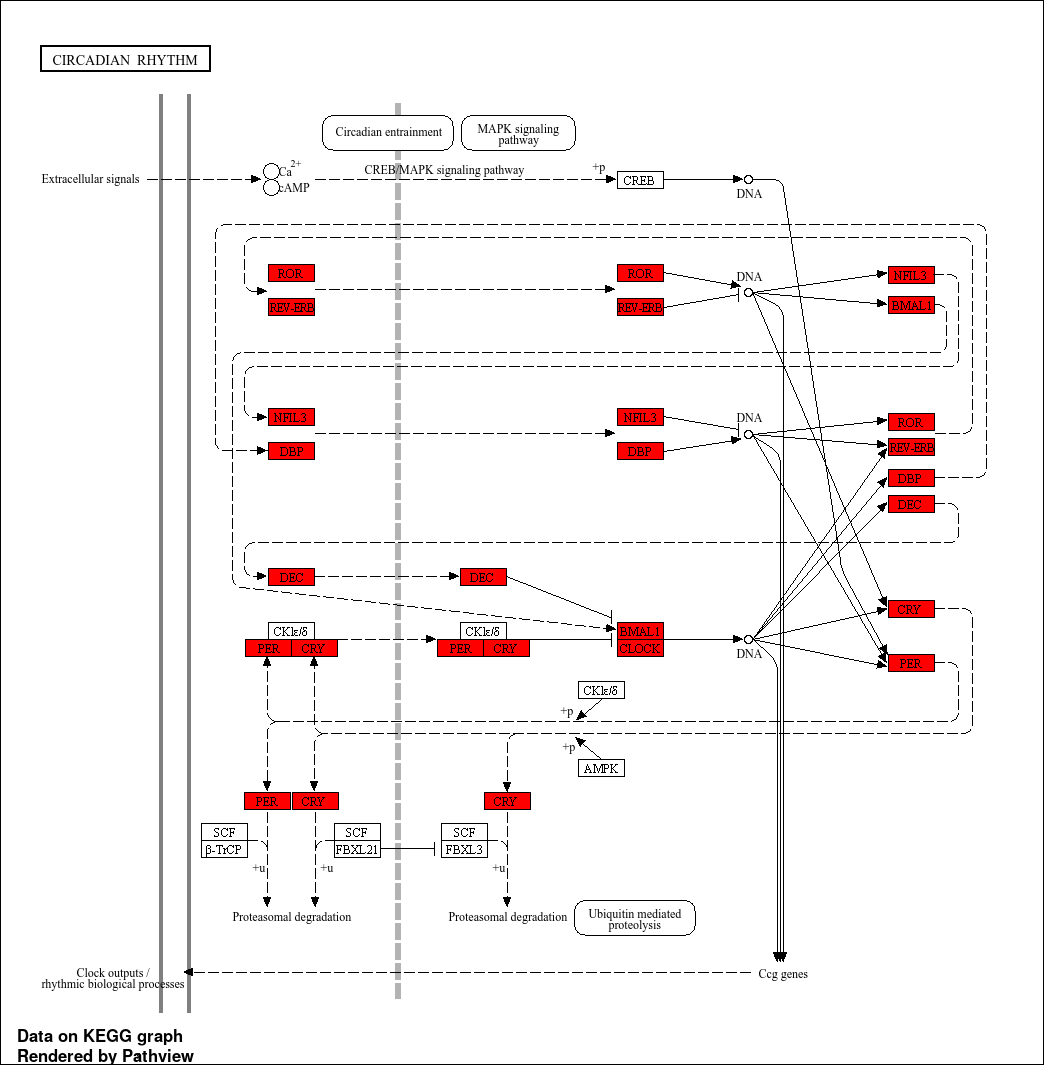

Supplement: Supplementary file 1 [file genes-15-01640-s001.zip › Supplementary File S5.png]
